# Asylum seekers’ perspectives on vaccination and screening policies after their arrival in Greece and The Netherlands

**DOI:** 10.1371/journal.pone.0226948

**Published:** 2019-12-26

**Authors:** Christina Louka, Elizabeth Chandler, Adelita V. Ranchor, Hans Broer, Spyros Pournaras, Sofanne J. Ravensbergen, Ymkje Stienstra

**Affiliations:** 1 Department of Internal Medicine, University Medical Center Groningen, University of Groningen, Groningen, Netherlands; 2 ESCMID study group for infections in travelers and migrants, Basel, Switzerland; 3 Department of Health Psychology, University Medical Center Groningen, University of Groningen, Groningen, Netherlands; 4 Vluchtelingenwerk, Netherlands; 5 Laboratory of Clinical Microbiology, ‘ATTIKON’ University Hospital, School of Medicine, National and Kapodistrian University of Athens, Athens, Greece; Public Health England, UNITED KINGDOM

## Abstract

**Introduction:**

Europe has been dealing with an increasing number of refugees during the past 5 years. The timing of screening and vaccination of refugees is debated by many professionals, however refugees’ perspectives on health issues are infrequently taken into account. In this study, we aimed to investigate asylum seekers’ perspectives on infectious diseases screening and vaccination policies.

**Materials and methods:**

Interviews were conducted in Greece and the Netherlands. Asylum seekers and recently arrived refugees were approached and informed with the help of interpreters; consent forms were acquired. The survey focused on demographic data, vaccination status, screening policies and prevention of infectious diseases.

**Results:**

A total of 61 (43 male, 70.5%) refugees (30 Afghanis, 16 Syrian, 7 Erithrean) were interviewed. Mean age was 35.2 years (SD 13.5) and 50% had received primary or secondary education, while 24.6% received none. Median time after arrival in Greece and the Netherlands was 24 months (IQR 8.5–28). 44 out of 61 (72.1) participants were willing to be vaccinated after arrival in Europe, 26 preferred vaccination and screening to be performed at the point of entry. The need for screening and vaccination was perceived higher amongst participants in Greece (100% vs 43.3%) due to living conditions leading to increased risk of outbreaks.

**Conclusion:**

Participants were willing to communicate their perspectives and concerns. Screening and vaccination programs could be more effective when implemented shortly after arrival and by involving asylum seekers and refugees when developing screening and vaccination interventions.

## Introduction

Political turmoil, warfare and instability, specifically in Middle Eastern and African countries, during the past few years have led to an increase in refugees and asylum seekers entering Europe. In the Netherlands, 15.410 asylum seekers have arrived between January 2018 and October 2018 [1]. In Greece, 29.404 refugees have arrived overseas between January 2018 and November 2018, while the estimated arrivals through mainland are over 12.000 for the same period. Asylum seekers mostly originate from Syria, Iraq and Afghanistan [2].

Crowded conditions in refugee camps or settlements and the lack of systematic medical care during their transnational journey, may contribute to the dispersion of infectious diseases among this vulnerable group. Therefore, vaccinations and infectious diseases screening programmes in the hosting countries aim to protect public health by preventing dissemination of infectious diseases [3,4].

A recently published study showed a variety of approaches towards vaccination of both adult and child migrants across the EU/EEA. In addition, most guidance is not always migrant specific and the available guidance is frequently not applied in practice [5]. A recent systematic review documenting the effectiveness of European approaches towards migrant screening revealed that in European countries migrants are screened mostly in vertical disease programs, commonly active or latent tuberculosis, or both.

Although recommendations have been made regarding refugees’ vaccination policies, a WHO report in 2017 revealed that less than a third of countries have migrant specific guidelines on immunisation in their national programmes and documented differences in policy, guidance, and implementation [6]. Moreover, several studies have shown a high prevalence of micro-organisms expressing antimicriobial resistance (AMR) in the asylum seeker population. Specifically regarding asylum seekers in the Netherlands, it has been shown that prevalence of such microbes is higher compared to the general Dutch population [7], supporting AMR screening at hospital admission.

Furthermore, the optimal timing for implementation of screening and vaccination activities is frequently debated [8,9]. The stressful and dependent situation for asylum seekers upon arrival is considered to complicate free decision making by asylum seekers on health issues. Limited data is available regarding asylum seekers’ perspectives on such policies.

In this study, we aimed to investigate the perspectives of refugees and asylum seekers regarding screening and vaccination policies in order to obtain helpful information on the optimal strategy and especially its timing within the migration process. This knowledge could be used in decision making regarding optimal screening and vaccination programmes and their implementation.

## Materials and methods

### Study setting

Interviews were conducted in Greece and in the Netherlands in order to include asylum seekers at different stages of their asylum seeking journey and document possible differences regarding their perspectives on vaccination and screening policies.

### Greece

Interviews were conducted, between May and June of 2018, at the Structure of Welcoming and Hosting of Refugees, Schisto, Athens. Refugee camps in Greece are under the authority of the Greek Ministry of Migration. In order to conduct the study, all necessary forms were sent to the Ministry and we were granted special permission to access the camp in Schisto. The refugee camp has been operational since February 2016 and has a capacity of approximately 1000 refugees. The Structure functions under the administrative and financial supervision of the Ministry of Migration. At the time of the study, 880 refugees were hosted at the camp. Asylum seekers residing at the structure were approached by our team with the help of an interpreter at the communal places of the structure.

### Netherlands

The interviews were conducted in various locations including the offices of ‘Vluchtelingenwerk’, a non-governmental organization, between October of 2017 and February of 2018. Other locations were the tuberculosis center and the department of Internal Medicine of the UMCG. These departments often provide medical care for asylum seekers. Patients were approached during admission or during outpatient visits.

### Approach and data analysis

Asylum seekers and refugees were approached with the help of an interpreter and the study was thoroughly explained to the participants. All professional interpreters were officially trained and certified to communicate and work with asylum seekers. Interviews were conducted in English, Dutch, or any other language with assistance by a professional interpreter in person or by phone.

Data was anonymously recorded using the Qualtrics survey program. The interviews were carried out until data saturation was noted independently by two researchers. Data was analyzed using the statistical software SPSS (SPSS Inc., version 23.0, Chicago, Illinois) and Excel (Microsoft Excel 2016). Descriptive statistics were used to calculate percentages. Open coding was used to analyze the qualitative information from the open-ended questions on the questionnaire.

Throughout the paper Greece and the Netherlands will be referred to as the hosting countries.

### Questionnaire development

A semi structured questionnaire was designed to investigate the perspectives of asylum seekers with regard to vaccination and screening in the hosting countries. The questionnaire was composed for the purpose of the study under the guidance of an experienced health psychology researcher (A.V.R.) in University Medical Center Groningen (UMCG), because no standard questionnaire was available for this topic.

The questionnaire was divided into three main parts; the first part was vaccination oriented, the second part focused on screening of multidrug-resistant organisms (MDRO) and tuberculosis and the third part involved questions regarding infectious diseases screening in general followed by a brief discussion on this topic. Considering different educational backgrounds of the participants and the complexity of terms like vaccination and screening, various verbal approaches by the interpreters were used, in order to simplify the questions. In addition, we used visual aids, such as photos of medical equipment used in screening and vaccination, i.e. syringes, swabs and x-rays.

The estimated duration of the interview was approximately 50 minutes. Prior to the start of the study, five pilot interviews were conducted. Potential ambiguities were identified and the questionnaire was revised accordingly. An online tool was used (Qualtrics) during the interviews, to enter the data and record the answers. Participants were reminded of the option not to answer specific questions of the survey in case they did not want to or could not.

### Inclusion criteria

Asylum seekers or refugees that arrived in the hosting countries, at least 4 months prior to the study, were included in the study to allow interviewees to form an opinion based on the experiences in the first months after arrival. In addition, only asylum seekers or refugees who arrived in the hosting countries after 1/4/2014 were included, so that interviewees would be able to recollect their experiences with vaccination and screening procedures. Asylum seekers younger than 18 years old were excluded. Information on children vaccination was obtained by interviewing their caretakers.

### Ethics

In Greece the study was approved by the Hellenic Centre for Disease Prevention and Control and the Ministry of Migration (protocol number ΚΠ 15161/2017-02/11/2017, 3/3908/03.04.2018). In the Netherlands, this study was evaluated by the Ethics committee and was waived in accordance with Dutch Legislation University Medical Centre Groningen, METc number non-WMO METc 2017/294. A written informed consent was obtained by all included participants. All participants were given the option to withdraw from the study at any given moment without having to give an explanation and were reassured that any potential withdrawal would have no impact in their health care and asylum status.

## Results

### General characteristics

Table 1 and Table 2 show the general characteristics of the total of the study group and by country were the interviews were conducted, respectively. In total, eight asylum seekers refused to participate, with the main reasons being timing (n = 3), exhaustion because of fasting during Ramadan (n = 2), cultural limitations to talk to a male interpreter without their spouse present (n = 2), and lack of opinion on the discussed subjects (n = 1).

**Table 1:** General characteristics of the total of the 61 participants interviewed in both Greece and the Netherlands.

	Number of interviewees (n = 61)
Sex (male %)	43 (70.5)
Age (SD)	35.2 (13.5)
Number of months in hosting country median (IQR))	24.0 (8.5–28.0)
**Country of origin (%)**	
Afghanistan	30 (49.2)
Syria	16 (26.2)
Eritrea	7 (11.5)
Others*	8 (13.1)
**Educational level (%)**	
No education	15 (24.6)
Primary education	8 (13.1)
Secondary education	17 (27.9)
Bachelor’s/master’s	21 (34.4)
**Profession (%)**	
Construction worker	11 (18.0)
Health care worker	10 (16.4)
Teacher	6 (9.8)
Carpenter	5 (8.2)
Seamstress	4 (6.6)
Others	25 (41.0)
**Asylum granted (%)**	37 (60.7)

**Table 2:** General characteristics of the participants that were interviewed by country in which the interviews were conducted.

	Greece (n = 31)	Netherlands (n = 30)
Sex (male %)	23 (74.2)	20 (66.6)
Age in years (SD)	34.1 (13.3)	37.2 (14.1)
Number of months in hosting country, median (IQR))	24 (9–27)	25 (8.5–37.25)
**Country of origin (%)**		
Afghanistan	29 (93.5)	1 (3.3)
Syria	0	16 (53.3)
Eritrea	0	7 (23.3)
Iraq	1 (3.2)	1 (3.3)
Iran	1 (3.2)	0
Others	0	5 (16.7)
**Educational level (%)**		
No education	15 (48.4)	0
Primary education	4 (12.9)	4 (13.3)
Secondary education	7 (22.6)	10 (33.3)
Bachelor’s/master’s	5 (16.1)	16 (53.3)
**Profession (%)**		
Construction worker	8 (25.8)	3 (10.0)
Carpenter	5 (16.1)	0
Seamstress	3 (9.7)	1 (3.3)
Health care worker	1 (3.2)	9 (30.0)
Teacher	2 (6.4)	4 (13.3)
Others	9 (29.0)	13 (43.3)
**Asylum granted (%)**	11 (35.5)	26 (86.6)

In total, 61 (former) asylum seekers and recently arrived refugees were included in the study. 31 of the interviews were conducted in Greece. The majority of participants originated from Afghanistan and Syria, while nine participants orginated from Sub-Saharan countries. The most commonly used languages during the interview were Farsi (47.5%), Arabic (16.4%) and English (14.1%). All interviews in Greece were conducted in person with the assistance of professional interpreters. In the Netherlands all interviews were conducted in person, 14 of which without the help of a professional interpreter, in English(n = 8) or in Dutch (n = 6), while the remaining 16 interviews were conducted with the help of a professional interpreter over the phone.’

### Part A: Vaccination data and perspectives

#### Vaccinations in adults

53 out of the 61 participants (86.9%) had been vaccinated in their country of origin according to the national vaccination schedule. Only 22 out of the 61 (36.1%) participants were asked about their vaccination status by official authorities, health care workers or NGOs upon arrival in the hosting countries. 12 out of 61 subsequently received additional vaccinations (influenza (n = 2), polio (n = 1), tetanus (n = 1), hepatitis B (n = 1), unknown (n = 7)). Vaccination was mostly performed by Non-Governmental Organizations (NGO, n = 3) and National Healthcare Employees (n = 4).

#### Vaccinations in children

34 out of 61 (55.7%) participants had children under their care upon arrival in the hosting countries, of which 24 were asked regarding the children’s vaccination status. 31 out of 34 (91.2%) participants mentioned that the children had been vaccinated in their country of origin. 27 reported that the children received additional vaccinations in the hosting countries. The most frequently mentioned vaccinations were MMR (n = 3), DTP (n = 3), measles (n = 2) and mumps (n = 1). 13 could not recall which vaccines were given to the children. Children were mainly vaccinated by NGOs (n = 10), public health care facilities (n = 6), or a doctor at the asylum centre (n = 5).

#### Perspectives on vaccination

When asked regarding necessity of vaccination, all 31 participants interviewed in Greece perceived the need of vaccination as of high importance, while only 13 out of the 30 participants interviewed in the Netherlands expressed the same opinion. Point of entry in Europe was considered as the optimal timing for vaccination (n = 26), followed by holding level (n = 9). Reasons given regarding the optimal timing for vaccination was ‘to protect ourselves’ (n = 21) and ‘to stop diseases’ (n = 8). According to the opinion of the participants, it is of no importance by which organization the vaccination is performed, as long as it is performed (n = 25). Other preferences for the organizations performing vaccination were the public health care system (n = 11) and NGOs (n = 8).

#### Willingness and necessity of vaccination

In Greece, all 31 participants were willing to be vaccinated. In the Netherlands, 13 out of 30 were willing to be vaccinated. In order to have a better understanding on the perceived importance of vaccination by the participants, we included a question with specific amounts of money and whether the participants were willing to pay them in order to be vaccinated. When asked if participants were willing to pay €10,- for vaccination, 26 out of 31 responded positively in Greece, and 13 out of 30 responded positively in the Netherlands. When asked if participants were willing to pay €200, 12 out of 31 in Greece and only 1 out of 30 in the Netherlands responded positively.

55 participants considered increasing the vaccination rate among asylum seekers to be necessary. Explanations given for the wish to increase general vaccination was ‘for protection’(n = 13), ‘for prevention’ (n = 11), ‘for vulnerable population’ (n = 8) and ‘for overall health improvement’(n = 6).

#### Perspectives on promotional work

55 out of 61 participants considered promotional work on vaccination useful to increase the vaccination rate among asylum seekers. 29 participants emphasized the importance of increased educational activities such as seminars/presentations within the hosting facilities, while 8 preferred distribution of written informative material and 4 preferred outreach programmes.

### Part B Screening of infectious diseases

#### Hospital admissions and screening for MDRO

13 participants had experienced a hospital admission in the hosting countries, mostly at the department of pulmonary diseases (n = 5) and surgery (n = 4). During hospital admission, eight asylum seekers communicated with the medical staff with the help of professional interpreters.

Screening for MDRO was performed in three participants. One of them was informed regarding the rationale behind the screening. Nobody had any comments on how the MDRO screening could be improved.

#### Screening for tuberculosis and scabies

24 participants reported to have been screened for tuberculosis by X-Ray on arrival in the hosting countries. Eight participants were aware of the procedure prior to entering the hosting country, of which four were informed by friends or family members. The remaining four participants were informed by other resources, such as leaflets distributed in the camps and media. Two participants considered the TB screening as a negative experience. One of them was experiencing physical pain due to other health problems during the screening, while the other one reported the experience as negative due to lack of privacy especially when performed by a medical professional of the opposite sex.

Regarding scabies, 19 reported to have been screened for scabies on arrival in the hosting countries, mainly in Greece (n = 17). The general experience was described as satisfactory (n = 18), with the exception of one participant who indicated that not all of his clothes were returned after they were washed. 17 interviewees considered the screening to be necessary in order to decrease the burden of scabies. No further comments were made by the interviewees in order to improve the screening for tuberculosis and scabies.

### Part C: Other perspectives on infectious disease screening

53 participants considered screening for infectious diseases useful during the asylum procedure. Main reasons included prevention of infectious diseases among the asylum seeker population (n = 14), protection of their own health (n = 11), important for the health of both asylum seekers and non-asylum seekers population (n = 11), overall health improvement (n = 8) and the vulnerable aspect of the asylum seekers population (n = 7). Seven participants opted not to answer this question.

Participants preferred to expand infectious diseases screening policy for asylum seekers in order to include hepatitis B (n = 34), hepatitis C (n = 31) and HIV (n = 30). The majority of the participants were particularly concerned regarding sexually transmitted diseases (STDs) and would like themselves and other asylum seekers to be screened for STDs. Point of entry in Europe was considered as the optimal timing (n = 42) for such screening, followed by holding level (n = 6) and country granting asylum (n = 2). Participants expressed concern regarding asylum seekers’ sexual health even after arrival in the hosting countries. A recurring theme during the interviews was the conception that different culture and social behavior in the hosting countries could lead to more liberal sexual behavior and extra marital sex, without the knowledge how to protect one’s health.

When asked to elaborate and further comment on infectious diseases screening and vaccination implementation, the participants mainly focused on the importance of efficient systematic medical care (*‘good health is above all’*, ‘*first comes health*’) and infectious diseases control (‘*need to protect ourselves and others’*), while one of the participants used a rather relevant Afgani proverb, *‘when one sheep is sick then all sheep are sick’*. The main complaint expressed was insufficient medical care and/or understaffed structures (n = 9). Seven suggested that more information on health care and infectious diseases should be available. Particularly in Greece, four of the participants expressed concerns regarding scabies outbreaks and skin disorders and emphasized the burden of disturbing symptoms of such diseases (‘*children have a really hard time with the scratching and pain’*). In the Netherlands, participants did not report this scabies burden, possibly as a result of the scabies intervention program [10]. However, one of the participants indicated that not all of his clothes were returned after they were washed and he said that this had happened to others. Moreover, he had to stay in disposable overalls, while his clothes were being washed, as part of the intervention program for scabies and louse-borne relapsing fever [11] for longer than necessary, which he experienced as stigmatizing. 34 had no additional comments.

## Discussion

In this study, we aimed to investigate asylum seekers’ perspectives on existing vaccination and screening policies in Greece and the Netherlands. We interviewed 61 asylum seekers originating from various different countries, mainly being Afghanistan and Syria. The majority of them described not having a negative experience with the screening and vaccination programmes and considered these policies of great importance for the well-being of asylum seeker population and public health.

In most European countries, vaccination policies for asylum seekers mainly focus on children [5]. However, several studies have shown low vaccination coverage among adult asylum seekers [12–14]. A Dutch study that included mostly asylum seekers from Syria, Iran, Iraq and Afghanistan showed insufficient protection against specific preventable diseases. Adults younger than 25 years showed the lowest measles seroprevalence [15]. In our study, the majority of participants underlined the defaults regarding adult vaccination and were open to supplementary vaccination. However, participants in the Netherlands were less supportive of further vaccinations. This difference could be explained by the different time points within the asylum seeking process. Greece functions as one of the transit countries while the Netherlands serves as a recipient country. Asylum seekers often have to face multiple health checks and experience different national vaccination policies throughout their journey. By the time they reach their final destination, their health care priorities may have changed.

Timely implementation of vaccination and screening policies is currently recommended by WHO and UNHCR [16, 17]. A study from Sweden demonstrated awareness among asylum seekers regarding the benefits of timely screening, as participants expressed concern over the health risk posed by their living conditions and potential delay of screening appointment [18]. In accordance, in our study, the majority of the participants preferred screening and vaccination policies to be implemented at point of entry in Europe. Participants expressed concerns regarding the increased risk of infectious diseases when people coming from different countries live closely together in centres and camps.

A systematic review on AMR among migrants in Europe demonstrated high prevalence of AMR carriage and AMR infection in migrants and concluded that implementing protocols for the prevention and control of AMR is necessary to ensure migrant health [19]. During the second part of the interview, asylum seekers were asked whether they had been admitted to a hospital, and if yes, whether they had been screened for MDROs. In Greece, as expected, none of the people that were admitted were screened for MDROs since there is no national MDRO screening policy regarding hospitalized asylum seekers. In the Netherlands, half of the asylum seekers that were admitted, reported they had been screened.

The majority of asylum seekers and children in need of additional vaccination or medical care, reported NGOs as the health provider, and such organisations were chosen as the preferred provider when asked. Political and social debate on which health provider is responsible for vaccination, screening and general medical care of asylum seekers, in European hosting countries, have led to an increased involvement of NGOs and volunteers in migrant health. NGO employees and volunteers do not necessarily have any special training or formal links with the national health-care system. Thus, linking NGOs with national healthcare systems in order to avoid discrepancies and optimize referral strategies could be challenging [20].

During the final part of the interview, asylum seekers were asked to give their perspectives regarding further screening on infectious diseases such as HIV and Hepatitis B and C. The majority of the participants were particularly concerned regarding STDs. Asylum seekers are often uneducated or misinformed regarding safe sexual practices and prevention of STDs [21, 22].

The study was conducted in limited refugee camps and structures that we were granted access to, in Greece and the Netherlands. Subsequently, not all asylum seekers had the same probability to be included. Furthermore, distribution of demographic characteristics of the participants depended on the population composition at the time of the study. The number of invited participants depended on the documented feedback and interviews were carried out until data saturation was noted. Access to conduct the interviews in other asylum settings and in other time periods, may have yielded extra information. Another limitation of the study was an increased ratio of men to women participants. Furthermore the number of participants originating from Sub-Saharan countries was small, leading to a possible gap in our results regarding their perspectives.

A strength of this study was the diversity of the study population regarding country of origin and educational level. Another strength was the different structures we visited in order to recruit the asylum seekers for the interview, an aspect that contributed to the diversity of the study population. Moreover, by conducting the study both in Greece and in the Netherlands, participants were at a different stage of their journey at the time of the interview. The interviews in Greece were conducted at an early stage of the asylum seekers’ transnational journey while the interviews conducted in the Netherlands represent the perspectives of asylum seekers who are in the final stage of the asylum seeking process.

It has been proposed to implement screening and vaccination policies as a two parted action plan, with the first part mainly taking place at arrival in the temporary hosting country and the second part at their final recipient country [23, 24]. A similar two-step has the potential plan to resolve the gap in vaccination among adult asylum seekers. Furthermore, alteration of screening policies in order to be in line with ECDC recommendations. Regarding STDs, existing screening policies for infectious diseases could be expanded in order to include screening for HIV, Hepatitis B and C. In our study, the need of vaccinations and screening was perceived lower amongst the participants in the Netherlands. Implementation of programs including vaccination and screening after reaching the asylum granting country may therefore be complicated by a switch in health care priorities of the asylum seekers.

## Conclusions

To conclude, participants were willing to communicate their perspectives and concerns, and expressed a positive attitude towards vaccination and screening, understanding the rationale behind those policies for infection prevention and protection of public health. Our findings emphasize the need to include asylum seekers in the decision making of screening strategies. Based on the results, point of actions could be: (i) implementation of educational outreach programmes regarding screening, vaccination and safe sex practices, (ii) implementation of screening and vaccination programs will likely be more successful when the asylum seekers’ need is perceived the highest, which is soon after arrival, (iii) include asylum seekers in the decision making on screening and vaccination strategies, (iiii) potential use of an open, easy to access online platform for communication between policy makers and asylum seekers that could provide valuable information.
